# Transporters MRP1 and MRP2 Regulate Opposing Inflammatory Signals To Control Transepithelial Neutrophil Migration during Streptococcus pneumoniae Lung Infection

**DOI:** 10.1128/mSphere.00303-18

**Published:** 2018-07-05

**Authors:** Andrew Zukauskas, Randall J. Mrsny, Paula Cortés Barrantes, Jerrold R. Turner, John M. Leong, Beth A. McCormick

**Affiliations:** a Department of Microbiology and Physiological Systems, University of Massachusetts Medical School, Worcester, Massachusetts, USA; b Department of Pharmacy and Pharmacology, University of Bath, Claverton Down, Bath, United Kingdom; c Department of Pathology, Brigham and Women’s Hospital, Boston, Massachusetts, USA; d Department of Molecular Biology and Microbiology, Tufts University School of Medicine, Boston, Massachusetts, USA; Providence Portland Medical Center

**Keywords:** hepoxilin A3, MRP1, MRP2, neutrophil, PMN, *Streptococcus pneumoniae*, pneumococcus

## Abstract

Streptococcus pneumoniae remains a source of morbidity and mortality in both developed and underdeveloped nations of the world. Disease can manifest as pneumonia, bacteremia, and meningitis, depending on the localization of infection. Interestingly, there is a correlation in experimental murine infections between the development of bacteremia and influx of neutrophils into the pulmonary lumen. Reduction of this neutrophil influx has been shown to improve survivability during infection. In this study, we use *in vitro* biotinylation and neutrophil transmigration and *in vivo* murine infection to identify a system in which two epithelium-localized ATP-binding cassette transporters, MRP1 and MRP2, have inverse activities dictating neutrophil transmigration into the lumen of infected mouse lungs. MRP1 effluxes an anti-inflammatory molecule that maintains homeostasis in uninfected contexts, thus reducing neutrophil infiltration. During inflammatory events, however, MRP1 decreases and MRP2 both increases and effluxes the proinflammatory eicosanoid hepoxilin A3. If we then decrease MRP2 activity during experimental murine infection with S. pneumoniae, we reduce both neutrophil infiltration and bacteremia, showing that MRP2 coordinates this activity in the lung. We conclude that MRP1 assists in depression of polymorphonuclear cell (PMN) migration by effluxing a molecule that inhibits the proinflammatory effects of MRP2 activity.

**IMPORTANCE** Streptococcus pneumoniae is a Gram-positive bacterium that normally inhabits the human nasopharynx asymptomatically. However, it is also a major cause of pneumonia, bacteremia, and meningitis. The transition from pneumonia to bacteremia is critical, as patients that develop septicemia have ~20% mortality rates. Previous studies have shown that while neutrophils, a major bacterium-induced leukocyte, aid in S. pneumoniae elimination, they also contribute to pathology and may mediate the lung-to-blood passage of the bacteria. Herein, we show that epithelium-derived MRP1 and MRP2 efflux immunomodulatory agents that assist in controlling passage of neutrophils during infection and that limiting neutrophil infiltration produced less bacteremia and better survival during murine infection. The importance of our work is twofold: ours is the first to identify an MRP1/MRP2 axis of neutrophil control in the lung. The second is to provide possible therapeutic targets to reduce excess inflammation, thus reducing the chances of developing bacteremia during pneumococcal pneumonia.

## Abstract (precis)

Streptococcus pneumoniae is a Gram-positive bacterium that normally inhabits the human nasopharynx asymptomatically. However, it is also a major cause of pneumonia, bacteremia, and meningitis. The transition from pneumonia to bacteremia is critical, as patients that develop septicemia have ~20% mortality rates. Previous studies have shown that while neutrophils, a major bacterium-induced leukocyte, aid in S. pneumoniae elimination, they also contribute to pathology and may mediate the lung-to-blood passage of the bacteria. Herein, we show that epithelium-derived MRP1 and MRP2 efflux immunomodulatory agents that assist in controlling passage of neutrophils during infection and that limiting neutrophil infiltration produced less bacteremia and better survival during murine infection. The importance of our work is twofold: ours is the first to identify an MRP1/MRP2 axis of neutrophil control in the lung. The second is to provide possible therapeutic targets to reduce excess inflammation, thus reducing the chances of developing bacteremia during pneumococcal pneumonia.

## INTRODUCTION

Despite the availability of vaccines, antibiotics, and improved hygienic advancements, bacterial pneumonia remains a demanding worldwide medical challenge. The Centers for Disease Control and Prevention (CDC) estimate that Streptococcus pneumoniae (pneumococcus), the most common bacteria associated with community-acquired pneumonia, causes ~400,000 pneumonia cases in the United States and leads to ~35,000 deaths annually. Of those presenting with pneumococcal pneumonia, 30% develop bacteremia, a condition with mortality rates close to 20% (1, 2).

A hallmark of pneumonia pathophysiology is the recruitment of polymorphonuclear cells (PMNs) (or neutrophils) to the pulmonary lumen via extravasation and transepithelial transcytosis. Although PMN recruitment serves initially to clear invading bacteria, it also contributes directly to lung injury and pulmonary dysfunction (3–5). Studies have indicated that transepithelial PMN migration can actually mediate bacterial blood infiltration (6–10).

To better understand mechanisms regulating PMN influx during pneumococcal infection, we previously examined host mediators of S. pneumoniae-induced PMN migration. Transepithelial leukocyte migration during pneumococcal infection required the lipid chemoattractant hepoxilin A_3_ (HxA_3_), an eicosanoid synthesized from arachidonic acid via 12-lipoxygenase (12-LOX) and secreted by lung epithelial cells (9). Pharmacological inhibition or genetic ablation of 12-LOX profoundly decreased PMN influx into the lungs of S. pneumoniae*-*infected mice and resulted in both uniform survival and reduced bacteremia during an otherwise lethal pulmonary challenge (9). Thus, 12-LOX-dependent production of HxA_3_ and subsequent PMN transepithelial migration appear to be required for high-level bacteremia.

Most information regarding HxA_3_-induced PMN emigration is derived from studies involving intestinal Gram-negative bacterial infection (11–14). At the intestinal surface, HxA_3_ apical epithelial secretion requires the ATP-binding cassette (ABC) transporter multidrug-resistance-associated protein 2 (MRP2; also known as ABCC2 or c-MOAT) to establish a chemotactic gradient targeting PMNs to sites of inflammation (12, 15). Although ABC transporters were originally identified as contributors to multidrug resistance due to their capacity to extrude cytotoxic drugs, emerging reports suggest that they play roles in host defense and immune regulation (16–22). To date, few studies have identified ABC transporters that mediate proinflammatory activity in the lung during bacterial infection.

Herein, we demonstrate that ABC transporters MRP1 and MRP2 are diametrically expressed at the apical epithelial surface and actively efflux substrates that control PMN migration. Characterizing this relationship, MRP1 effluxes substrates that suppress PMN transmigration; MRP1 apical expression greatly diminishes upon S. pneumoniae infection. In contrast, MRP2 becomes highly enriched on the epithelial apical surface during pneumococcal infection and promotes PMN migration. Overall, these findings reveal a paradigm that epithelial cells at the lung mucosal surface act as critical sensors that can determine when to initiate PMN transmigration in response to a proinflammatory stimulus such as S. pneumoniae and when to maintain a noninflammatory state.

## RESULTS

### Survey of MRP expression patterns during Streptococcus pneumoniae infection.

To study inflammatory responses during infection with S. pneumoniae, we have established an *in vitro* model of PMN migration using polarized monolayers of NCI-H292 (H292) cells (23–25). We used this model to examine the profiles of multidrug resistance proteins (MRPs) that differ in mRNA or protein expression during S. pneumoniae infection. Whether analyzing whole-cell protein (see Fig. S1A and B in the supplemental material) or mRNA (Fig. S1C), little change was observed. Of note, MRP1 decreased slightly during infection at both the mRNA and protein level. No other values reached 10% difference among MRP2, MRP4, or MRP5. MRP3 and P-glycoprotein were not detected at the basal state and during infection at the protein or mRNA level (data not shown).

10.1128/mSphere.00303-18.1 FIG S1  MRP inquiry during infection. NCI-H292 cells were infected with Streptococcus pneumoniae, and MRP profiles were initially generated by two different techniques: protein Western blots and mRNA RT-PCR quantification. (A) MRP1, -2, -3, -4, and -5 and P-glycoprotein (P-gp) were investigated for possible changes upon infection with Streptococcus pneumoniae via Western blotting, which showed slight reduction in MRP1 but no other strong changes. A representative blot from three separate experiments is shown. (B) Densitometry of Western blots (three separate experiments which each had a Western blot associated with it). The protein of interest was normalized to loading control (GAPDH), and fold changes comparing the values for infected-cell samples to uninfected buffer samples were calculated. (C) RT-PCR revealed a slight reduction in MRP1 and slight increases in MRP2 and MRP5 during pneumococcal infection. P-gp and MRP3 were not detected by any of these methods. Download FIG S1, PDF file, 1.2 MB. Copyright © 2018 Zukauskas et al. 2018 Zukauskas et al. This content is distributed under the terms of the Creative Commons Attribution 4.0 International license.

### MRP1 and MRP2 show inverse patterns of expression during S. pneumoniae infection.

Though there were few changes observed in mRNA and protein levels, it is documented that MRPs undergo critical posttranslational modifications that affect their subcellular localization (26, 27). HxA_3_ efflux draws PMNs to the site of infection, in this case to the epithelial cell apical surface. To that end, we sought to specifically examine changes in apical localization of our chosen proteins. We, therefore, selectively labeled the apical surface of infected epithelial surfaces with biotin and compared labeled proteins in infected and uninfected surfaces.

**FIG 1:**
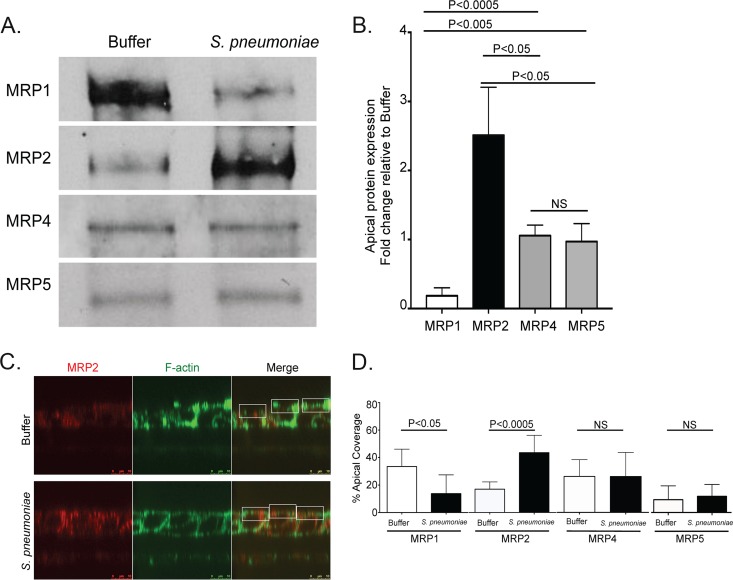
MRP1 protein on the apical surface reduces during infection with Streptococcus pneumoniae, while MRP2 increases. The apical surface examination of mock-infected or *S. pneumoniae* serotype 4 (TIGR4)-infected polarized H292 cells. The cells were treated with HBSS (Buffer) or infected (S. pneumoniae), washed, and allowed to rest at 37°C for 1 h postinfection. (A) Apical surfaces were then labeled with biotin and lysed. Samples were normalized to protein content against a BSA standard, exposed to streptavidin beads, and subjected to sodium dodecyl sulfate-polyacrylamide gel electrophoresis (SDS-PAGE). The blots were probed with primary antibodies for the selected proteins. A representative Western blot of apical biotinylation probing MRP expression is shown. (B) Densitometry of pooled Western blot samples from multiple experiments. Statistics calculated using two-tailed Student’s *t* test compared with MRP4 or MRP5 (*n* = 3). (C) Buffer-treated or infected cells were fixed and stained for MRP2 and F-actin. Immunofluorescence cross-section Z-stack images of F-actin were utilized to identify cellular borders and apical surface (green). The corresponding region of MRP2-stained Z-stack (red) was marked for the particular region of interest (white boxes) and calculated for the total apical coverage using ImageJ. (D) The calculated percentage of the area taken up by the indicated MRPs in panel C via calculations completed with ImageJ (see Materials and Methods). *P* values were calculated using two-tailed Student’s *t* test comparing the percentages for the given protein in uninfected and infected cells (*n* = 8). Values that are not significantly different (NS) are indicated. Quantification samples for biotinylation and immunofluorescence were taken from at least two separate infections with similar results.

Biotinylated apical MRP1 drastically decreased after infection with S. pneumoniae, while apical MRP2 increased (Fig. 1A and B). In comparison, surface-expressed MRP4 and MRP5 did not change (Fig. 1A and B). Using immunofluorescence microscopy, we confirmed that pneumococcal infection induced localized decreases in MRP1 and reciprocal increases in MRP2 (Fig. 1C and D). Importantly, we observed no change in MRP4 or MRP5 surface localization via immunofluorescence following pneumococcal infection. On the basis of these data, we focused our studies on potential modulation of MRP1 and MRP2.

**FIG 2:**
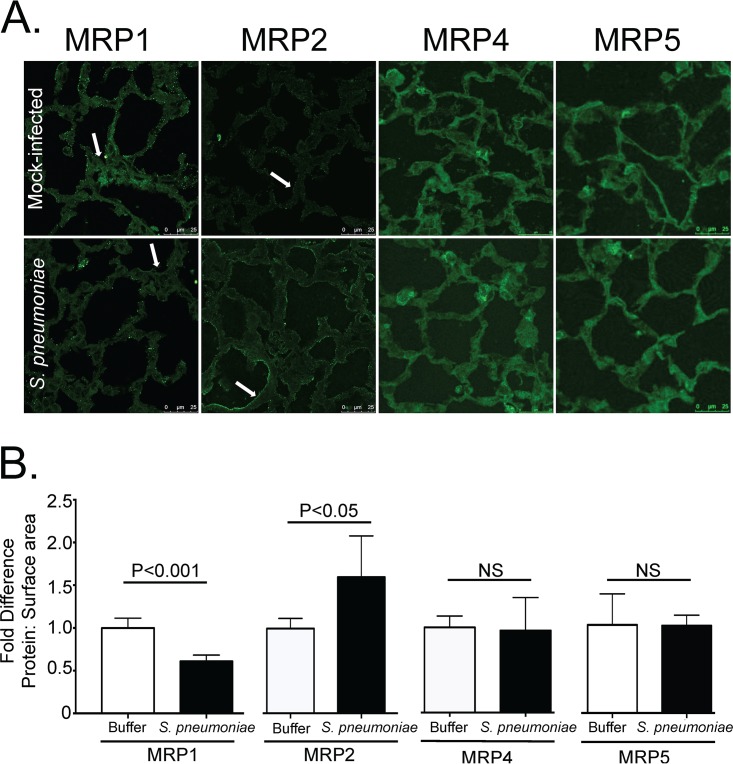
S. pneumoniae infection reduces MRP1 and increases MRP2 upon pulmonary infection in mice. Mice were infected via an intratracheal route with S. pneumoniae and sacrificed 2 days postinfection. The lungs were excised, reinflated, sectioned, and stained for MRP1, MRP2, MRP4, or MRP5. (A) Representative images from three different experiments. Arrows indicate points of interest. In particular, areas of similar density in MRP1-stained lungs appear to have reduced expression in infected lungs compared to uninfected lungs (arrow). MRP2 staining appears to increase drastically on the cell periphery during infection compared to mock-infected lungs (arrow). No such increases or decreases are observed for MRP4 and MRP5. (B) Quantification of staining. Antibody staining was quantified and normalized to surface area (measured by F-actin [not shown]). Fold differences of signal to surface area for each given antibody comparing infected (S. pneumoniae) to uninfected (Buffer) animals are shown. Values are expressed as fold increase or decrease compared to the values for uninfected samples.

To examine whether *in vitro* observations correlated with changes in *in vivo* MRP localization, C57BL/6J mice were infected with 2.5 × 10^5^ CFU of S. pneumoniae or mock infected with phosphate-buffered saline (PBS) alone. Two days postinfection, mice were sacrificed. The lungs were excised, sectioned, and probed to detect MRP1, MRP2, MRP4, and MRP5 via immunofluorescence (Fig. 2). Similar to our *in vitro* data, we saw decreases in the MRP1 signal and increases in MRP2 during pneumococcal infection. No such changes were observed with MRP4 or MRP5, pointing to a consistency in MRP modulation in both cell lines and the mouse (Fig. 2A and B).

### MRP2 controls PMN transmigration.

Given that MRP2 controls aspects of PMN transcytosis in intestinal epithelium (15), we sought to examine the ability of MRP2 to drive proinflammatory events in the context of S. pneumoniae infection. We previously showed that PMN transmigration into lung airways during pneumococcal infection required HxA_3_ (9). We also demonstrated that MRP2 upregulates during intestinal inflammation and that inhibition of MRP2 function suppresses PMN migration (15). It has not been shown, however, that MRP2 location or activity is affected by infection by Gram-positive bacteria, such as S. pneumoniae. On the basis of these previous findings, we examined whether pharmacological inhibition of MRP2 suppresses PMN transepithelial migration across pulmonary epithelium during pneumococcal infection.

To inhibit MRP2, cells were treated with probenecid, an MRP2 inhibitor (15, 28–30). *In vivo* probenecid is relatively well tolerated with death occurring in mice that received 1,600 mg/kg of body weight (31). It is possible for probenecid to affect other transporters, but more often these transporters need higher concentrations than that which is efficient to inhibit MRP2 (16, 32–34). In addition, probenecid has been shown to have some effects in inhibiting MRP1 (35); however, biotinylation data in Fig. 1 and 2 would indicate that MRP1 is already reduced during infection. Any effects inhibiting MRP1 would likely exacerbate, rather than curtail, PMN recruitment during infection. This idea will also be explored in more detail later in the manuscript.

**FIG 3:**
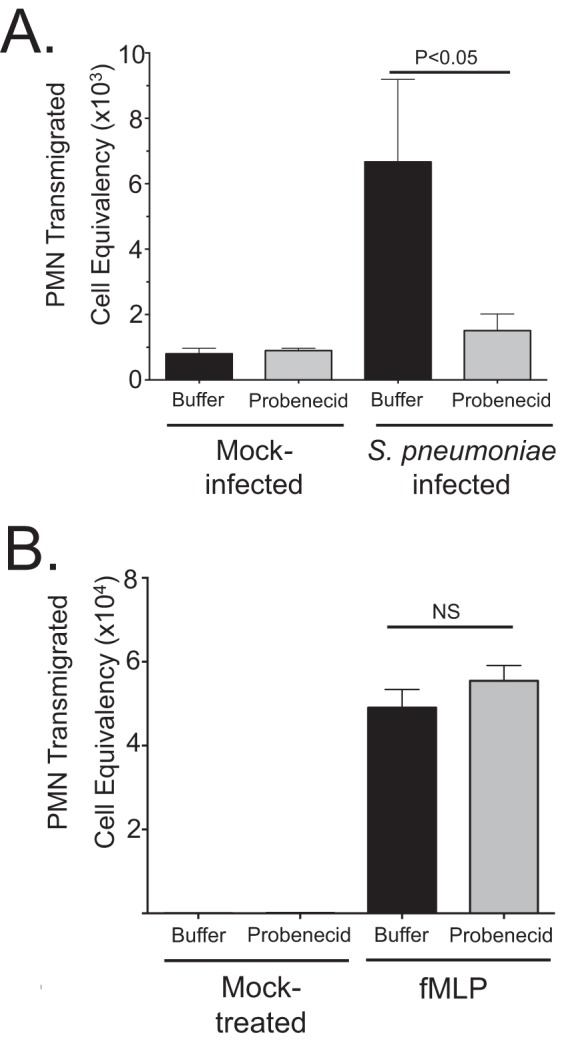
MRP2 inhibition via probenecid reduces PMN migration across polarized pulmonary epithelial cells. (A and B) Polarized NCI-H292 cells on filter membranes were incubated with 100 µM MRP2 inhibitor probenecid or mock treated for 1 h with PBS before apical infection with S. pneumoniae at an MOI of 10 (A) or the HxA_3_-independent bacterial product formyl-methionyl-leucyl-phenylalanine (fMLP) (B). For both panels A and B, mock-infected or mock-treated cells were exposed to HBSS for the treatment time period, and basolateral-to-apical migration of PMNs was quantified with a myeloperoxidase assay against a standard number of PMNs (see Materials and Methods). The results of one representative experiment from at least three experiments performed are shown. Statistical significance was calculated using two-tailed Student’s *t* test.

Cells were treated 1 h preinfection with probenecid and then subjected to infection and a PMN migration assay. Probenecid treatment had no effect on the basal PMN migration across uninfected cells but suppressed PMN transmigration across infected H292 monolayers by approximately threefold (*P* < 0.05) compared to mock-infected controls (Fig. 3A). However, when we used a different PMN chemoattractant that acts independently of the HxA_3_ pathway, formyl-methionyl-leucyl-phenylalanine (fMLP) (36, 37), we failed to observe any probenecid-related reduction in PMN migration (Fig. 3B). To ensure that the PMN transmigration was not due to pneumococcus-mediated epithelial cell apoptosis, we performed annexin V staining (Fig. S2) and found no significant increase in apoptosis during infection, consistent with previous reports (38).

10.1128/mSphere.00303-18.2 FIG S2  Apoptosis in wild-type cells. H292 cells were treated with HBSS, wild-type TIGR4 Streptococcus pneumoniae, or the cytotoxic compound staurosporine at 1 µM. There was no significant increase in annexin V staining in infected cells (black) compared to uninfected buffer control cells (white). Download FIG S2, PDF file, 0.04 MB. Copyright © 2018 Zukauskas et al. 2018 Zukauskas et al. This content is distributed under the terms of the Creative Commons Attribution 4.0 International license.

### MRP2 inhibition mitigates pulmonary PMN infiltration and bacteremia following lung challenge with S. pneumoniae.

S. pneumoniae intratracheal infection of C57BL/6J mice initially causes pneumonia and leads to bacteremia and, ultimately, to septicemia that causes death (39). As shown previously, reduced PMN migration can diminish bacteremia and improve survivability of pneumococcus-infected mice (6, 9, 10). With the knowledge that MRP2 inhibition diminishes PMN migration *in vitro*, we sought to test whether MRP2 function had subsequent impact on disease in our murine infection model, especially in the context of PMN transmigration.

Four sets of experiments were conducted: all involved mice that were pretreated with either PBS or probenecid at 1 mg/kg delivered via the trachea to inhibit MRP2 function. Previous studies of this specific model have indicated that maximal PMN recruitment occurs between 18 and 24 hours postinfection (10); therefore, two sets of mice were sacrificed at either 24 or 48 h postinfection to examine the leukocytes entering the lung via bronchial-alveolar lavage fluid (BALF) sample collection (see Materials and Methods). The third set was sacrificed 2 days postinfection to test for bacteremia and total lung burden. The fourth set was monitored for morbidity that would require sacrifice as dictated by our standard operating procedure (SOP) (see Materials and Methods).

**FIG 4:**
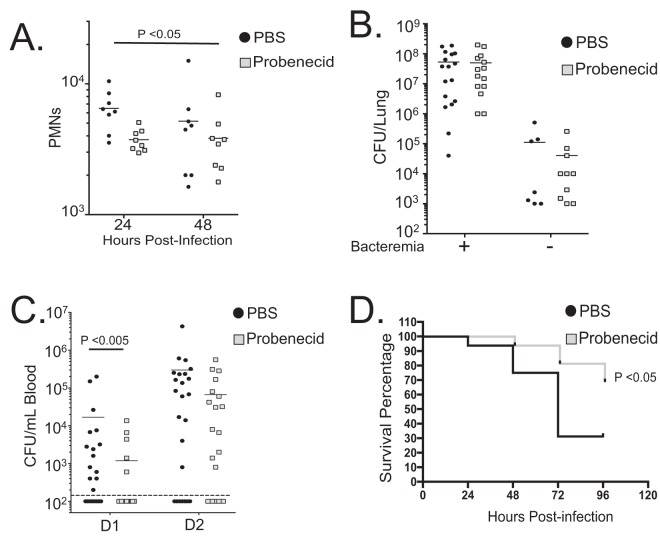
MRP2 inhibition mitigates pulmonary burden and bacteremia following lung challenge with S. pneumoniae. Using an *in vivo* model of PMN migration, we examined the results of MRP2 inhibition. C57BL/6 mice were treated with either PBS or probenecid 3 h before and 3 h after intratracheal application of 2.5 × 10^5^ S. pneumoniae. Four sets of mice were infected in this way. (A) Comparing the number of PMNs found in the lumen after isolating BAL fluid samples after 24 and 48 h postinfection. PMNs, identified as Ly6g-positive cells, were quantified by flow cytometry (*n* = 8). (B) Overall lung burden from mice sacrificed on day 2. Mice were sacrificed, lungs were excised and homogenized, and total bacterial burden was calculated using serial dilutions (*n* = 24 for each condition). The presence (+) or absence (−) of bacteremia is indicated as follows: +, mice had detected levels of bacteria in blood 48 h postinfection; −, no bacteria were detected in blood during tail vein bleeds. (C) Bacteremia, as measured by tail vein bleeds, from the cohorts in panel B (see Materials and Methods). Detected events of colony formation on day 1 (D1) and day 2 (D2) (*n* = 24) are shown. The broken line represents the limit of detection, and as such, values for mice without visible bacteremia were represented as 100 CFU/ml, just below this level of detection. In panels B and C, statistical significance was calculated using the Mann-Whitney test. (D) Survival experiment with the fourth set of mice. There were 16 mice in a group for each condition. Statistical significance was calculated using Mantel-Cox test and Gehan-Breslow-Wilcoxon test. Probenecid treatment consistently increased survival by approximately 30 to 40% during survival experiments and often delayed symptoms, such as lethargy. Mouse experiments were repeated, and similar results were observed in at least two different experiments.

Upon examining the contents of BAL fluid samples, we noted that probenecid treatment consistently reduced the number of PMNs on the first day postinfection by ~35% (Fig. 4A). This trend continued through 48 h postinfection, and the combined values of 24- and 48-h PMN infiltration showed significant differences as calculated using two-way analysis of variance (ANOVA). When measuring expression of pro- and anti-inflammatory cytokines from the BAL fluid samples, we noted that there appeared to be no substantial differences between probenecid-treated and mock-treated animals in any of the measured inflammatory cytokines at the times mentioned, with exception to a reduction in interleukin 10 (IL-10) in probenecid-treated mice at 24 h postinfection (Fig. S3A to D). We do not believe that this is a substantial physiological difference, as reductions or eliminations of IL-10 in S. pneumoniae-infected mice generally lead to greater infiltration of PMNs, not decreased infiltration (40). No other significant differences in T cells, macrophages, or dendritic cells were observed (Fig. S4A to C). Despite the reduction in PMNs, no differences were observed in pulmonary bacterial burden 48 h postinfection (sacrificed whether exhibiting morbidity or active and healthy [Fig. 4B]) or upon death during survival experiments (Fig. S4D).

10.1128/mSphere.00303-18.3 FIG S3  ELISA data show no significant proinflammatory differences. Three-milliliter BAL fluid samples from infected mice in Fig. 4A (and Fig. S4B to D—all samples are from the same eight mice). Both mock-treated (PBS) and probenecid-treated mice were infected, and BAL fluid was tested for TNF (A), keratinocyte-derived chemokine (KC) (the murine equivalent to IL-8) (B), macrophage inflammatory protein 1 (MIP-1) (C), and IL-10 (D). No significant differences were observed except for IL-10, as calculated by two-way ANOVA. Values were slightly lower than expected due to the elution of BAL fluid in 3 ml instead of the classic 800 μl to 1 ml. Download FIG S3, PDF file, 0.1 MB. Copyright © 2018 Zukauskas et al. 2018 Zukauskas et al. This content is distributed under the terms of the Creative Commons Attribution 4.0 International license.

10.1128/mSphere.00303-18.4 FIG S4  Pulmonary burden during survival experiments and *in vivo* granulocyte enumeration. (A to C) Additional leukocytes identified in the BAL fluid quantified by flow cytometry. These include T cells (Cd3^+^ cells) (B), macrophages (Cd11b^+^ Cd45^+^ Ly6g^−^ cells) (C), and dendritic cells (Cd11c^+^ Cd11b^−^ Ly6g^−^ cells) (D). Samples are from the same set of samples as that in Fig. 4A. Experiments were repeated with similar results. (D) Pulmonary burden was calculated using serial dilution of lung homogenates during survival experiments in Fig. 4, taken from mice that died. Like the previous bacteremia experiments, no difference in bacterial burden was observed at the endpoint of survival examinations. All statistical significances were calculated using Mann-Whitney test (for individual time points) as well as two-way ANOVA (combined time points). Download FIG S4, PDF file, 0.1 MB. Copyright © 2018 Zukauskas et al. 2018 Zukauskas et al. This content is distributed under the terms of the Creative Commons Attribution 4.0 International license.

In line with previously mentioned data supporting that reduction in infiltrating PMNs reduces bacteremia, probenecid-treated animals showed fewer numbers of mice exhibiting bacteremia and ~10-fold-lower CFU in blood during the first 24 h compared to PBS-treated mice (Fig. 4C). This trend continued at the 48-h time point, although it did not reach statistical significance. The resulting survival curves showed probenecid treatment improved survival by 30 to 40% consistently (Fig. 4D). Therefore, MRP2 inhibition via probenecid treatments was found to be effective at limiting PMN infiltration to the lung 24 h postinfection, corresponding to a reduction in bacteremia and increased survival. While it is tempting to speculate on the therapeutic potential of probenecid, in this experiment, this inhibitor is being used as an agent to block the functional effect of a biological target.

### MRP2 and MRP1 promote the secretion of proinflammatory and anti-inflammatory lipids, respectively.

**FIG 5:**
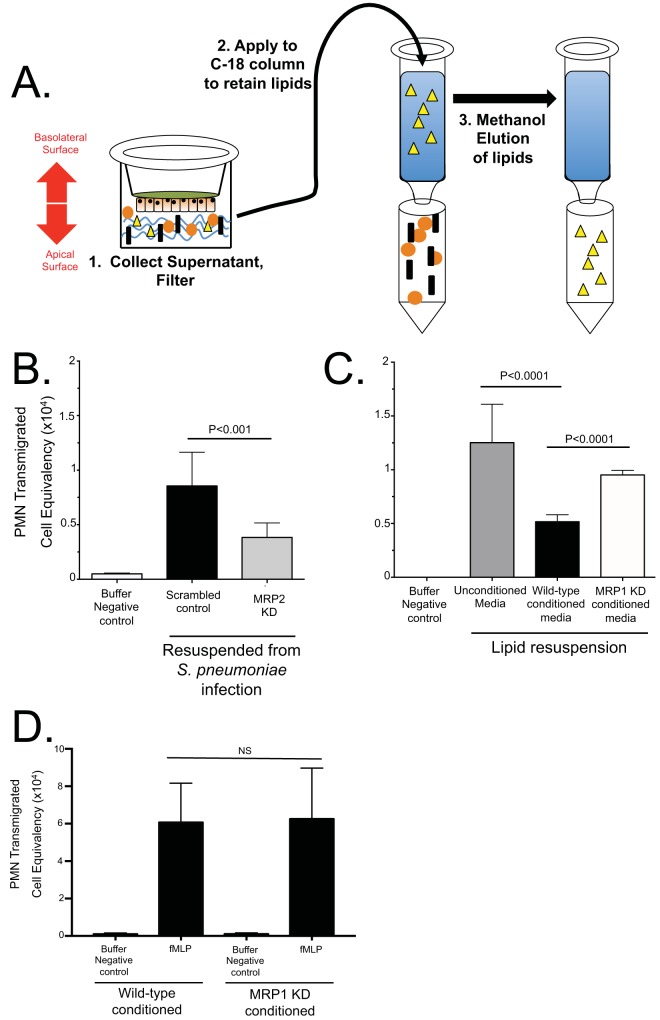
MRP2 and MRP1 promote the secretion of proinflammatory lipids and anti-inflammatory molecules, respectively. (A) Proinflammatory lipids were isolated from apical supernatants of pneumococcus-infected H292 monolayers. HBSS subjected to the apical surface of pneumococcus-infected H292 cells were enriched on C_18_ columns, which retain the proinflammatory lipids, and eluted in methanol for storage (see Materials and Methods). Yellow triangles represent proinflammatory lipids. (B) Lipids from MRP2-competent (Scrambled control) cells and MRP2-deficient (MRP2 knockdown [MRP2 KD]) cells were enriched and eluted with methanol. This methanol/lipid solution was evaporated under a constant stream of nitrogen and resuspended in HBSS to be used as PMN chemoattractant during a PMN migration assay through a monolayer of naive wild-type H292 cells. (C) Proinflammatory lipids isolated from wild-type cells infected with Streptococcus pneumoniae from panel A were resuspended with either unconditioned media, conditioned media from MRP1-competent scrambled-control cells (Wild-type conditioned media), or conditioned media from MRP1 knockdown cells (MRP1 KD conditioned media) and applied to the apical chamber of naive cells to act as a chemoattractant during a PMN migration assay. HBSS without any lipid acted as a negative control (Buffer Negative control). (D) MRP1-conditioned media failed to inhibit HxA_3_-independent PMN-migration produced using formyl-methionyl-leucyl-phenylalanine (fMLP). The values were not significantly different (NS) by two-tailed Student’s *t* test.

To better understand how MRP1 or MRP2 function could impact pneumococcus-induced PMNs, we generated H292 cell lines constitutively expressing small hairpin RNAs (shRNAs) targeting MRP1 or MRP2 expression (Fig. S5). Because it is known that MRP2 is able to efflux the proinflammatory molecule HxA_3_ in the intestines (15), we hypothesized that limiting MRP2 expression would reduce the amount of HxA_3_ effluxed during pneumococcal infection. As HxA_3_ is an eicosanoid, we began by assessing the properties of hydrophobic material(s) enriched from apical secretions of S. pneumoniae-infected polarized H292 cell monolayers. We used a previously described method (15) utilizing methanol elution of a C_18_ column to enrich bioactive lipid(s) following application of a pathogen to the apical surface of an epithelial monolayer (Fig. 5A). To reconstitute the lipid chemoattractant, the methanol-lipid solution was evaporated over a steady stream of compressed nitrogen and resuspended with Hanks balanced salt solution (HBSS). The isolated lipid-HBSS was then applied to the apical surface of naive H292 cells during a PMN transmigration assay. As expected, resuspended lipid isolated from uninfected cells yielded no PMN migration, indicating there is no chemoattractant produced by the cells without infection (Fig. 5B). When we applied resuspended lipid chemoattractant isolated from infected cells to the apical surface of H292 cells during a PMN transmigration assay, we induced PMN migration (Fig. 5B). Extracts isolated from H292 monolayers with reduced MRP2 expression were less effective at inducing PMN transmigration than H292 cells transfected with a scrambled shRNA sequence, in a manner similar to data reported in Fig. 3 for probenecid, indicating a direct correlation between MRP2 expression and PMN migration (Fig. 5B). To ensure that the process of lipid isolation enriches for HxA_3_, we performed a functional assay in which HxA_3_-containing lipids are treated with soluble epoxide hydrolase (sEh) after being resuspended in HBSS, as has been described (41). sEh treatment of HxA_3_ reduces the epoxide ring and results in the biologically inactive trioxilin form of the eicosanoid. As HxA_3_ is the only known lipid PMN chemoattractant that contains an epoxide ring, any resulting reduction in PMN transepithelial migration would largely be attributed to the reduction in HxA_3_. Consistent with this notion, sEh treatment resulted in a marked decrease in transepithelial PMN migration compared to the migration in mock-treated samples (Fig. S6).

10.1128/mSphere.00303-18.5 FIG S5  shRNA constructs result in knockdown of MRP1 and MRP2 expression. H292 cells were transfected with a control shRNA plasmid containing a scrambled construct, one of three MRP1 shRNA constructs, or one of three shRNA MRP2 constructs. Cells were selected under puromycin antibiotic selection and assessed via Western blotting. A representative Western blot of three lysis events is shown. Cultures with arrows indicate the shRNA pool selected for MRP1/MRP2 knockdown experiments. Download FIG S5, PDF file, 0.3 MB. Copyright © 2018 Zukauskas et al. 2018 Zukauskas et al. This content is distributed under the terms of the Creative Commons Attribution 4.0 International license.

10.1128/mSphere.00303-18.6 FIG S6  Soluble epoxide hydrolase reduces HxA3 bioactivity in lipid resuspension. Lipids isolated as shown in Fig. 5A were resuspended in HBSS and either mock treated or treated with soluble epoxide hydrolase (sEh) to test that the lipid resuspension was enriched for HxA_3_. Lipids were then applied to naive H292 cells to undergo a transepithelial PMN migration assay (see Materials and Methods). sEh treatment significantly reduced the amount of chemoattractant in the lipid resuspension solution (gray) compared to mock-treated samples (black), indicating that HxA_3_ is present as a major component of the lipid resuspension solution. *P* values were calculated using two-tailed Student’s *t* test. Download FIG S6, EPS file, 0.3 MB. Copyright © 2018 Zukauskas et al. 2018 Zukauskas et al. This content is distributed under the terms of the Creative Commons Attribution 4.0 International license.

On the basis of reduced MRP1 surface expression during pneumococcal infection, we further hypothesized that MRP1 is involved in an anti-inflammatory cascade that suppresses PMN transepithelial migration. To test this theory, we once again resuspended the isolated lipid chemoattractant from Fig. 5A to conduct a PMN migration assay. Instead of resuspending the chemoattractant with conventional HBSS, we conditioned HBSS with apical supernatants from either scrambled-control or MRP1-deficient knockdown (KD) cells. We expected MRP1 to secrete a molecule or molecules during basal, uninflamed states (when MRP1 was highest) that would directly inhibit PMN transmigration. Media conditioned with MRP1 competent cells, then, would reduce PMN migration. Media conditioned with apical supernatants from MRP1 KD cells would show no such inhibition.

When examining the results of conditioning the media with uninfected apical secretions, the resulting PMN migration with normal HBSS without isolated lipids induced no PMN migration, as expected (Fig. 5C). Lipids resuspended with media conditioned with wild-type, MRP1-competent cells inhibited PMN migration compared to lipids resuspended with unconditioned HBSS (Fig. 5C). When conducting a PMN migration with lipids conditioned with MRP1-deficient cells, we lost the inhibition observed with cells that had intact MRP1 expression (Fig. 5C). When we again used fMLP to examine the HxA_3_ dependence, we again failed to observe a reduction in PMN migration (Fig. 5D). In addition, we saw no changes in tumor necrosis factor (TNF-α), IL-8, CXC chemokine ligand 2 (CXCL2), or IL-10 signaling in MRP1 knockdown cells either before or after infection as measured by an enzyme-linked immunosorbent assay (ELISA) (Fig. S7). Thus, it appears that MRP1 inhibits HxA_3_-specific PMN migration *in vitro* and by reducing MRP1 expression, we diminished the inhibition on the PMN migration that we observed with wild-type cells.

10.1128/mSphere.00303-18.7 FIG S7  Neither MRP1 knockdown nor probenecid treatment significantly changes the ELISA profile of tissue culture cells within 1 h of infection. Wild-type (WT) (black circles), scrambled-control (SC) (open circles), MRP1 knockdown (MRP1) (triangles), and probenecid-treated (Prob) (gray squares) cells were subjected to ELISA sample collection. HBSS was incubated for 1 h postinfection in either mock-treated cells (Buffer) or Streptococcus pneumoniae-infected cells (S. pneumoniae) and collected from the apical surface for ELISA. No significant changes were observed for TNF-α (A), IL-8 (B), CXCL2 (C), or IL-10 (D) when comparing Scrambled-control cells to MRP1 knockdown cells or wild-type cells to any of the other conditions. Two separate runs were pooled together, and experiments were repeated with similar results. Download FIG S7, EPS file, 0.6 MB. Copyright © 2018 Zukauskas et al. 2018 Zukauskas et al. This content is distributed under the terms of the Creative Commons Attribution 4.0 International license.

## DISCUSSION

Lower respiratory tract infections are among the leading causes of death worldwide, and S. pneumoniae remains the deadliest bacterial agent causing this affliction. Infection of the lung with S. pneumoniae induces a variety of inflammatory responses, the pathological hallmark of which is massive influx of PMNs. In this role, PMNs are signaled to navigate to the site of infection and have the task of pathogen eradication. Outright neutropenia or elimination of PMN activity at this step is detrimental to the host, as a full adaptive immune reaction has not had time to activate, and the bacteria are unabated. However, unchecked, PMN recruitment lacking resolution culminates in massive tissue destruction and, ultimately, lung failure. Thus, one of the key challenges in treating lung inflammation lies in attenuating the inappropriate influx of PMNs without compromising the ability of the patient to fight normal infections. We envisage that a better understanding of the mechanisms underlying the regulation of PMN influx during pneumococcal infection is essential to designing improved therapies that dampen detrimental lung inflammation. This is a logical approach for S. pneumoniae, as this bacterium is encapsulated, countering PMNs as a critical host defense mechanism. Thus, the cost of host tissue damage as a result of PMN infiltration of the airway epithelium during pneumococcal infection significantly outweighs any benefit PMNs may have in defense of this particular infection.

**FIG 6:**
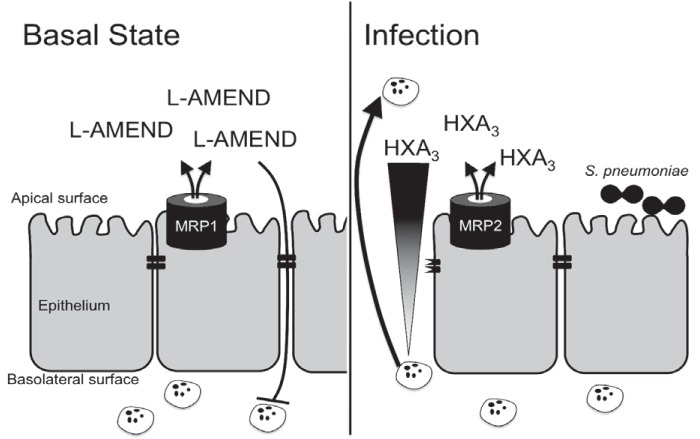
Epithelial MRPs assist in controlling pro- and anti-inflammatory states. As shown by this work, lung epithelium expresses large amounts of MRP1 and small amounts of MRP2 at basal (uninfected) states. MRP1 effluxes molecules with anti-inflammatory activity, here termed L-AMEND, which acts to suppress PMN migration. During infection with Streptococcus pneumoniae, MRP1 is reduced and MRP2 increases on the apical surface of the epithelium. MRP2 mediates release of the proinflammatory molecule hepoxilin A3 (HXA_3_), which creates a chemokine gradient to draw PMNs to the site of infection. By both reducing MRP1 and increasing MRP2, the epithelium works to maximize the transepithelial PMN migration; however, this PMN migration also disrupts epithelial tight junctions and can lead to infiltration of the intruding S. pneumoniae.

Although several studies have probed aspects of PMN recruitment to the airspace using a range of *in vitro* and *in vivo* models, many molecules mediating innate immune responses during pneumococcal pneumonia infection are redundant and/or dispensable for PMN recruitment (42–49). As shown herein, we have uncovered a system of checks and balances in which eukaryotic ABC efflux transporters facilitate the coordination of PMN transepithelial migration across lung epithelia in response to pneumococcal infection. We have found that expression of the efflux transporter MRP1 on the apical surface of the lung epithelium is high in a normal basal state. The action of MRP1 at this site is to maintain homeostasis through efflux of immunosuppressive bioactive molecules that we have termed L-AMEND (lung activity-modulating epithelial-neutrophil discourse). Under the same homeostatic state, expression of another efflux pump, MRP2, is quite low, reinforcing the anti-inflammatory arm of this pathway. However, upon introduction of a foreign invader, such as S. pneumoniae, expression of MRP1 is decreased, reducing the effective concentration of anti-inflammatory molecules at the site of infection. MRP1 reduction is accompanied by an increase in the apical expression of MRP2, which facilitates the efflux of the proinflammatory eicosanoid hepoxilin A_3_, a potent PMN chemoattractant that in turn, attracts PMNs to the site of infection/injury (Fig. 6).

Critically, while it is known that HxA_3_ is necessary in pneumococcus-induced PMN translocation (9) and lung infection (50), no studies have ever shown the link between MRP2-dependent HxA_3_ efflux and Gram-positive bacteria, highlighting the conservative nature of the HxA_3_/MRP2 axis. Additionally, we found that blockade of MRP2 by probenecid during S. pneumoniae infection profoundly decreased PMN influx into the lungs of infected mice and, in turn, reduced the extent of systemic bacteremia. Our discovery that MRP1 effluxes L-AMEND, which suppresses PMN migration, provides two potential and independent treatment strategies to reduce PMN migration and thus limit development of bacteremia: apical presentation of exogenous L-AMEND or local inhibition of MRP2. It remains to be seen as to what exactly makes up the components of the L-AMEND. When examining efflux supernatants from MRP1 knockdown cells or probenecid-treated cells, we saw no discernible differences in the conditioned milieu as examined by ELISA (see Fig. S7 in the supplemental material).

We chose an intratracheal administration of bacteria in our studies to highlight the transition from lung to blood infection. One of the major focuses of this study was to examine whether MRP2 inhibition would augment PMN infiltration to the lungs during infection. Secondary to this, we sought to confirm that the PMN infiltration correlated with the development of bacteremia over the course of infection. Intratracheal infection seemed the most direct route to test this, bypassing any bottleneck in intranasal application, and guaranteeing that bacteria enter the lung. Histology revealed that lung pathology does not appear different between probenecid-treated and PBS-treated animals (Fig. S8), inferring that all of the mice had equal opportunity to develop bacteremia. Because the infection is delivered almost directly to the lung in a bolus, it is reasonable that both cohorts would exhibit similar lung pathology, since the major difference between the two is induction of bacteremia and PMN infiltration, not the lung infection itself (9, 10). One explanation for these observations is that HxA_3_ efflux specifically controls initial PMN migration but is not necessarily critical for prolonged PMN migration, which appears to be dictated by leukotriene B4 release from PMNs themselves (41). In probenecid-treated mice, therefore, MRP2 blockade would lead to fewer paracellular openings and only slightly fewer PMNs but a significant reduction in the number of breakages by which bacteria pass through.

10.1128/mSphere.00303-18.8 FIG S8  Histological examination shows little difference between probenecid and PBS treatment during infection. Mouse lungs were excised, sectioned, and underwent H&E staining. A pathologist examining for signs of infection and immunological response then scored slides in a blind manner. Although pathology scores worsened when comparing 24 and 48 h postinfection, no statistically significant differences were observed between probenecid- and PBS-treated animals. Lungs were excised either 24 h postinfection (24 PI) or 48 h postinfection (48 PI). PBS- and probenecid-treated animals without infection were scored and grouped whether sacrificed 24 or 48 h after mock infection. *n* = 4 for infected animals, *n* = 3 for PBS- and probenecid-treated mock-infected animals for each time point. Abbreviations: Prob, 1 mg/kg intratracheal probenecid treatment; *Sp*, Streptococcus pneumoniae-infected animals. Download FIG S8, EPS file, 0.3 MB. Copyright © 2018 Zukauskas et al. 2018 Zukauskas et al. This content is distributed under the terms of the Creative Commons Attribution 4.0 International license.

While our studies are the first to identify a push/pull interaction between MRP1 and MRP2 in PMN migration, there is another study examining MRP1 that presents an interesting paradox. Schultz et al. previously reported that global *Mrp1* knockout mice were protected during intranasal pneumococcal (serotype 3) infection in a leukotriene B4 (LTB_4_)-dependent manner, whereas wild-type littermates succumb to infection (51). At 48 h postinfection, extracellular leukotriene C4 (LTC_4_) was lower in BALF specimens from *Mrp1*^*−/−*^mice than in BALF specimens from wild-type mice. Intracellular LTC_4_, understandably, was found to be higher, leading to the conclusion that release of LTC_4_ was inhibited by the elimination of murine MRP1 (51). In a separate study, intracellular LTC_4_ retention was suggested to be cytotoxic (19). Because PMNs express MRP1, a global knockout would yield MRP1-deficient PMNs. This could lead to PMN apoptosis and reduced numbers, exactly the observation in the Schultz study 48 h postinfection. With this explanation, it would appear that the previously reported *Mrp1* knockout data do not refute our stated results. Additionally, this is yet another communication reinforcing that fewer PMNs responding to bacterial infection actually can improve survival in S. pneumoniae infection.

There remains the possibility that epithelium-derived LTB_4_ could influence PMN migration; however, it has been shown that LTB_4_ released by cells during *in vitro* PMN transmigration is almost exclusively derived from PMNs (41), eliminating the possibility that the epithelium is coordinating PMN migration with LTB_4_ in our MRP1 knockdown studies. To ensure that our infected epithelia do not undergo LTC_4_-induced apoptosis, we tested annexin V staining postinfection in MRP1-deficient epithelium compared to control cells (Fig. S9) and found no differences, indicating that any increases in PMN migration associated with MRP1 deficiency are not caused by epithelial cytotoxicity.

10.1128/mSphere.00303-18.9 FIG S9  MRP knockdown does not increase apoptosis in S. pneumoniae-infected cells. H292 cells with control constructs, MRP1 shRNA, or MRP2 shRNA underwent staining for apoptosis postinfection. There were no significant increases in apoptosis between scrambled control and knockdown cells during the staining procedure. *P* values were calculated using two-tailed Student’s *t* test. Download FIG S9, EPS file, 0.3 MB. Copyright © 2018 Zukauskas et al. 2018 Zukauskas et al. This content is distributed under the terms of the Creative Commons Attribution 4.0 International license.

Our hypothesis that MRP1/L-AMEND dictates an anti-inflammatory state while MRP2/HxA_3_ assists in proinflammatory activity incorporates the concept that epithelial cells (through regulation of ABC efflux transporters) act as sensors that integrate signals to determine when and to what level to incite PMN transmigration (Fig. 6). Thus, a steady-state, noninflamed condition is established that limits inappropriate inflammatory responses but is poised to respond to pathogens, such as S. pneumoniae. Although this PMN response is critical in controlling the infection at hand, prolonged PMN activation also has deleterious effects on health, highlighting a cost-benefit relationship for the host. These findings regarding the nature of this anti-/proinflammatory balance could provide clinical targets for therapeutic control of dysregulated respiratory innate inflammatory responses that ultimately allow for containment of the infection but simultaneously dampen detrimental lung inflammation.

## MATERIALS AND METHODS

### Cell culture.

The human mucoepidermoid pulmonary cell line, NCI-H292 (H292), was cultured with RPMI 1640 (catalog no. 11875-093; Gibco) supplemented with 10% fetal calf serum (FCS) and subcultured using 0.05% trypsin-EDTA (catalog no. 25300-054; Gibco). Transmigration cells were prepared on 24-well inverted semipermeable membranes (catalog no. 3421; Costar) and grown to confluence. Cells for RNA/protein assays were grown to confluence on six-well membranes. MRP1, MRP2, and scrambled-control knockdown cell lines were developed by chromosomal integration of pLOK.1 small hairpin RNA (shRNA) constructs in wild-type H292 cells, selected with puromycin, and confirmed by Western blotting.

### Bacteria.

Streptococcus pneumoniae serotype 4 (TIGR4) was provided by John Leong and Andrew Camilli (Tufts School of Medicine). Bacteria were streaked overnight on 5% sheep blood tryptic soy agar (catalog no. p-1100; Northeast Laboratory Services) and grown at 37°C and 5% CO_2_ for 14 to 20 h. On the day of infection, bacteria were resuspended with Todd-Hewitt-yeast medium and supplemented with Oxyrase (catalog no. OB-0100; Oxyrase, Inc.) (5 µl/ml culture). Cultures grew to ~5 × 10^8^ CFU/ml. *In vitro* infections were diluted in Hanks balanced salt solution (HBSS) (catalog no. 14025-092; Gibco). Mouse infection solutions were resuspended using filter-sterilized phosphate-buffered saline (PBS).

### RT-PCR.

Confluent H292 cells on six-well membranes were rinsed with HBSS and infected apically with TIGR4 (multiplicity of infection [MOI] of ~10) for 1 h. The cells were washed with HBSS, and RNA was collected using Qiagen RNeasy kits (catalog no. 74106; Qiagen) following the suggested protocol. cDNA was obtained using Qiagen QuantiTect following the manufacturer’s instructions. Reverse transcription-PCR (RT-PCR) data were obtained using Sybr green with custom primers for each transporter (in triplicate) normalized to expression of glyceraldehyde-3-phosphate dehydrogenase (GAPDH).

### Transmigration.

Transmigrations were conducted as previously described (52) using H292 cells. Probenecid (catalog no. P8761-25G; Sigma) was diluted in HBSS before being solubilized with NaOH and brought to pH 7.4 with HCl. Probenecid treatment at 100 µM was completed 1 h prior to infection. H292 cells were infected apically with wild-type TIGR4 (MOI of ~10) for 1 h and washed. Human PMNs were isolated from healthy donors using methods previously published (15). Freshly isolated PMNs were applied to the basolateral chamber of infected membranes and allowed to migrate. PMN migration was quantified using a myeloperoxidase assay. *N*-Formyl-methionyl-leucyl-phenylalanine (fMLP) was purchased from MP Bioscience (catalog no. 151170) and used at a final concentration of ~150 nM during PMN migrations. Soluble epoxide hydrolase was purchased from Cayman Chemical (catalog no. 10011669) and mixed in solution with resuspended lipid extracts at 100 µg/ml for 1 h prior to being exposed to naive cells during a PMN migration assay as previously described (41).

### Biotinylation.

H292 cells were grown on six-well transmembranes to confluence, rinsed with HBSS, and infected with TIGR4 (MOI of ~10) apically. The cells were washed and biotinylated as previously described (53, 54). The cells were lysed and passed through 26 1/2-gauge needles, spun, and applied to streptavidin beads (catalog no. 20347; Thermo Fisher) or saved in Tricine sample buffer (recipe from Bio-Rad [catalog no. 161-0739]). Biotinylated-bead samples were incubated at 4°C overnight, washed, incubated at 40°C for 20 min in Tricine sample buffer, then run on 4 to 20% TGX protein gel. The proteins were transferred onto nitrocellulose membranes, blocked, and incubated with antibodies against the following: MRP1 (ab24102; Abcam), MRP2 (ab3373; Abcam), MRP3 (ab3375; Abcam), MRP4 (ab56675; Abcam), MRP5 (ab77369; Abcam), P-glycoprotein (P-gp) (catalog no. 517310; EMD Millipore), and GAPDH (MAB374; EMD Millipore). Horseradish peroxidase (HRP)-conjugated secondary antibodies were used to visualize Western blots.

### Mouse infections.

C57BL/6J mice were purchased from Jackson Laboratories. For lung immunofluorescence images, mice were anesthetized with isoflurane and mock infected with PBS or infected with 50 µl of TIGR4 intratracheally (~2.5 × 10^5^ CFU/infection). Infections progressed for 48 h, and mice were sacrificed. The lungs were reinflated with 1 ml of 1:1 PBS/OCT mixture, frozen in OCT, and processed. For histological studies, mice were sacrificed, and the lungs were reinflated and fixed with PBS containing 10% formalin for a minimum of 24 h. The lungs were processed, sectioned, and hematoxylin and eosin (H&E) stained. Jerrold Turner and associates scored pathology. CFU was calculated via lung homogenization and serial dilution plating.

For probenecid studies, mice were pretreated 3 h prior to infection with 1 mg of probenecid per kg of body weight, diluted in PBS and applied intratracheally. Mice were mock infected with PBS or infected with 2.5 × 10^5^ CFU TIGR4, after which probenecid was administered 3 h postinfection. For bacteremia, 10 µl of blood was removed via tail vein lancet and diluted in anticoagulant for serial plating. ELISA samples and leukocyte panels were generated using a modified BALF isolation protocol (55). One-milliliter samples of PBS were applied to the mouse lung via the trachea three times and spun in a centrifuge at 2,000 rpm. Supernatant was frozen for ELISA experiments, and cells were washed and went to fluorescence-activated cell sorting (FACS) processing. Following red blood cell lysis, cells and Fc receptors (catalog no. 101319; BioLegend) were blocked in PBS containing 0.5% fetal bovine serum (FBS). Staining was achieved via incubation with Cd11b (catalog no. 101236; BioLegend), Ly6G (catalog no. 127618; BioLegend), Cd11c (catalog no. 117308; BioLegend), Cd3 (catalog no. 100204; BioLegend), or CD45 (catalog no. 103130; BioLegend) and run on a MACSquant analyzer. Populations were analyzed via FlowJo. ELISA kits were obtained from R&D Systems (catalog no. DY453-05, DY452-05, DY217B-05, and DY410-05), and assays were performed using the manufacturer’s instructions.

For survival curves, mice were sacrificed when exhibiting three of the following symptoms within 6 h, according to our standard operating procedure (SOP): lethargy, shivering, loss of more than 10% body weight, and fur ruffling. In all survival experiments, animals showed no signs of pathology after 96 h of infection. Repeated experiments going to 168 h postinfection showed similar results. Survival curve statistics were calculated using the Mantel-Cox test and Gehan-Breslow-Wilcoxon test. All experiments were completed with approval of University of Massachusetts IACUC 1905.

### Immunofluorescence.

Immunofluorescent H292 samples were grown on inverted membranes and either mock infected or infected with TIGR4 at an MOI of ~10 apically prior to fixation with 4% paraformaldehyde. The cells were washed, blocked with 3% bovine serum albumin (BSA), and permeabilized with 0.01% Triton X-100. The samples were stained with primary antibodies to MRP1 (sc-7773; Santa Cruz), MRP2 (sc-5770; Santa Cruz), MRP3 (ab3375; Abcam), MRP4 (ab56675; Abcam), or MRP5 (ab77369; Abcam). Secondary antibody incubations were with Alexa Fluor 488/phalloidin 569 (catalog no. A12380; Life Technologies) or Alexa Fluor 568/phalloidin 488 (catalog no. A12379; Life Technologies). Images were taken with a Leica SP-5 confocal microscope, and calculations were completed using ImageJ. Images were converted to 8 bits, the threshold was set for a given protein, and the percent area in a given region of interest was calculated. All corresponding uninfected and infected images were infected, stained, and imaged on the same day to reduce confounding factors and standardize the apical coverage calculations.

Mouse lung sections were fixed with 4% paraformaldehyde, blocked with 3% BSA, and permeabilized with 0.01% Triton X-100. Slides were incubated with the indicated primary antibody, washed, and incubated with 1:1,000 dilutions of the above secondary antibodies. Increases or decreases of signal were calculated using ImageJ to assign an arbitrary signal with the parameters described with H292 immunofluorescence. Fold differences in Fig. 2B were calculated by quantifying signal for specific antibodies and normalizing to surface area according to phalloidin staining. These ratios were averaged, and the buffer average was set at a ratio of 1. All samples were then standardized to the buffer average for each given antibody, and fold increases or decreases were reported.

### Lipid isolation.

H292 and MRP2 knockdown (KD) cells were grown and infected as described above under “Biotinylation.” HBSS applied after infection was collected, debris was removed via centrifugation, and solution was applied to a C_18_ solid-phase extraction column (catalog no. 52604-U; Supelco). Lipids were eluted using methanol and stored at −80°C for up to 1 month.

To analyze activity, lipid-methanol solutions were dried under constant nitrogen stream and resuspended in prewarmed HBSS. HBSS-lipid was applied to the apical surface of inverted naive H292 cells, and PMN migrations were completed as described above.

MRP1 inhibition was tested using prewarmed HBSS applied to the apical surface of control or MRP1 KD cells for 5 h, producing conditioned media. Lipid-methanol solutions from above were dried and resuspended with unconditioned HBSS, conditioned HBSS from MRP1-competent cells, or conditioned MRP1 knockdown media. Migrations were then conducted as described above.

Human ELISA kits were obtained from R&D Systems (DY210-05, DY217B-05, DY276-05, and DY208-05), and assays were completed using the manufacturer’s instructions. To collect samples, six-well filter insert plates were seeded with H292 cells and grown to confluence. The cells were equilibrated in prewarmed HBSS and either mock treated or infected with Streptococcus pneumoniae at an MOI of 10 on the apical surface for 1 h. The cells were then washed, and 1 ml HBSS was placed on the apical surface for 1 h for sample collection.

### Apoptosis.

For apoptosis, inverted H292 cell constructs were infected with TIGR4 at an MOI of 10 or treated with staurosporine (sc-3510a; Santa Cruz) as a positive control for 1 h. The cells were washed and allowed to rest at 37°C in HBSS for an additional 2 h. The cells were lifted using 0.05% trypsin-EDTA, washed, and stained with annexin V-fluorescein isothiocyanate (FITC) and propidium iodide (sc-4252 AK; Santa Cruz). Staining was visualized, and the cells were enumerated using a MACSquant analyzer and FlowJo. Three constructs per condition were pooled, and data shown are the summation of three different infections.
