# Supplementary figures and images for: Transporters MRP1 and MRP2 Regulate Opposing Inflammatory Signals To Control Transepithelial Neutrophil Migration during Streptococcus pneumoniae Lung Infection
Source: mSphere. 2018 Jul 5;3(4):e00303-18. doi: 10.1128/mSphere.00303-18 (PMC6034076; doi:10.1128/mSphere.00303-18)

# Supplemental Fig. 1

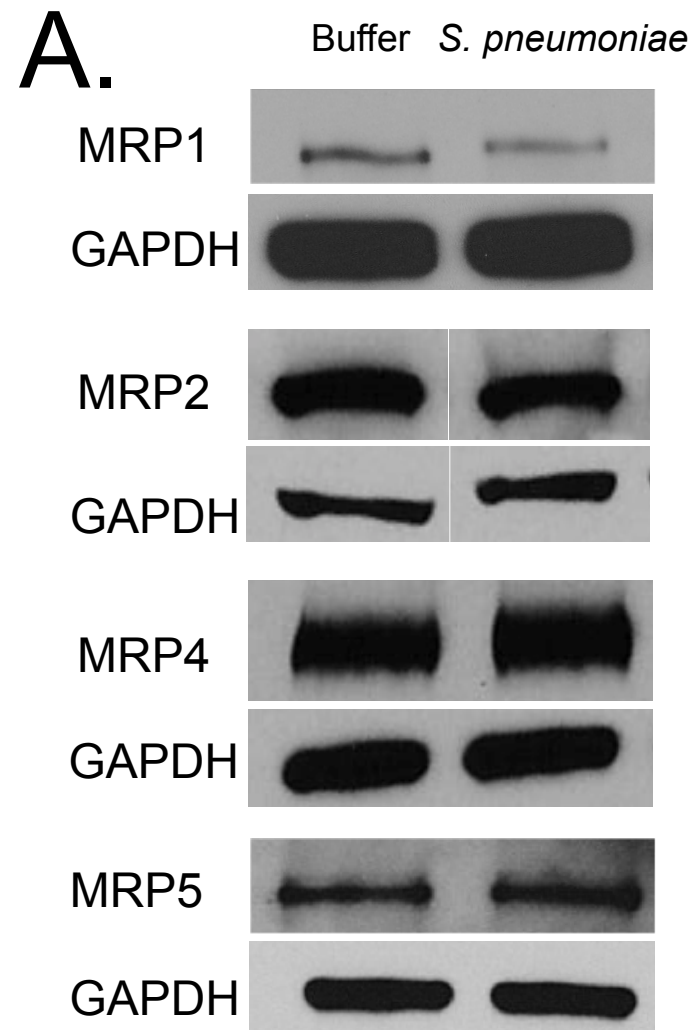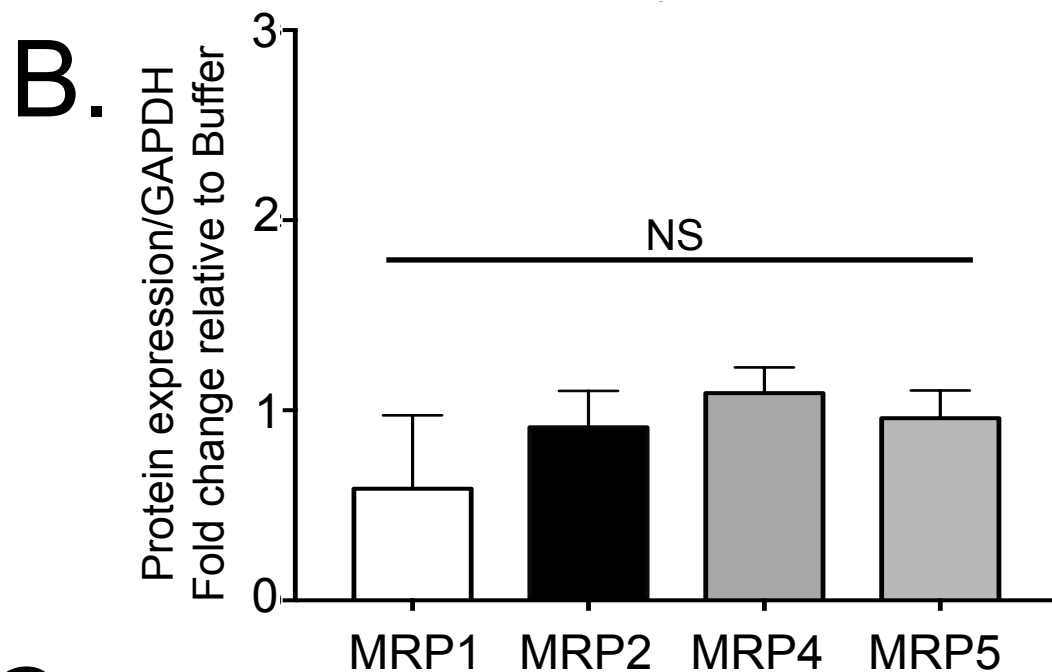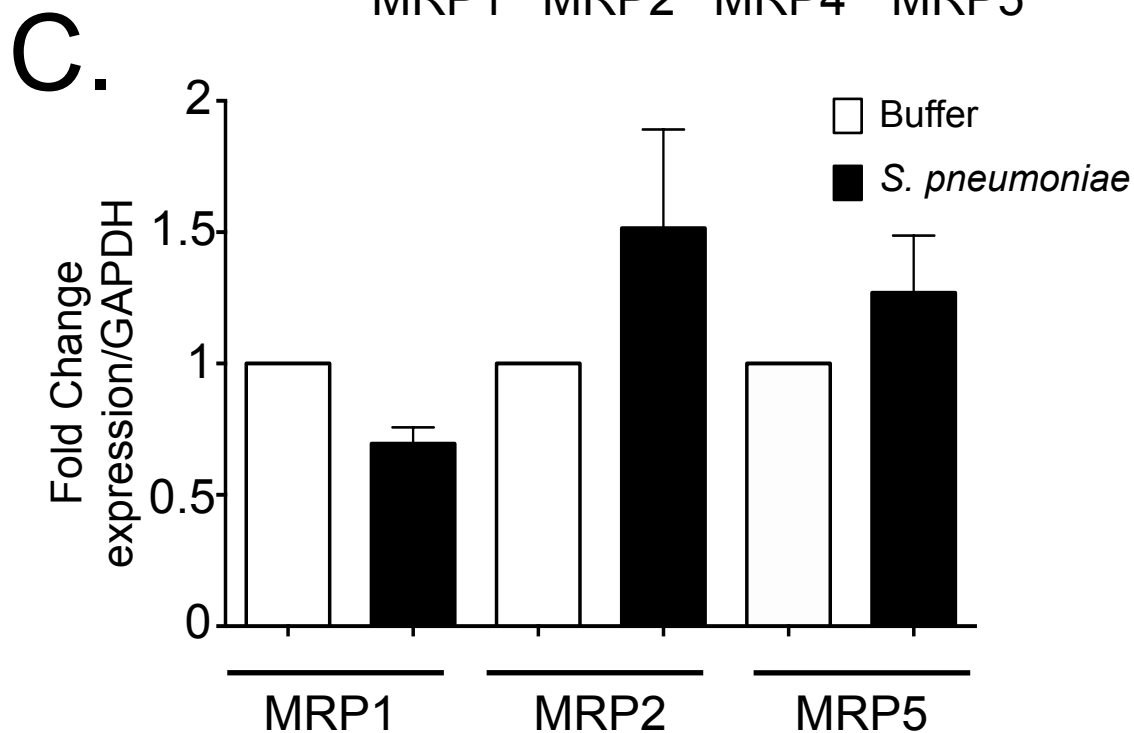

Supplement: FIG S1 [file sph003182582sf1.pdf]

# Supplemental Fig. 2

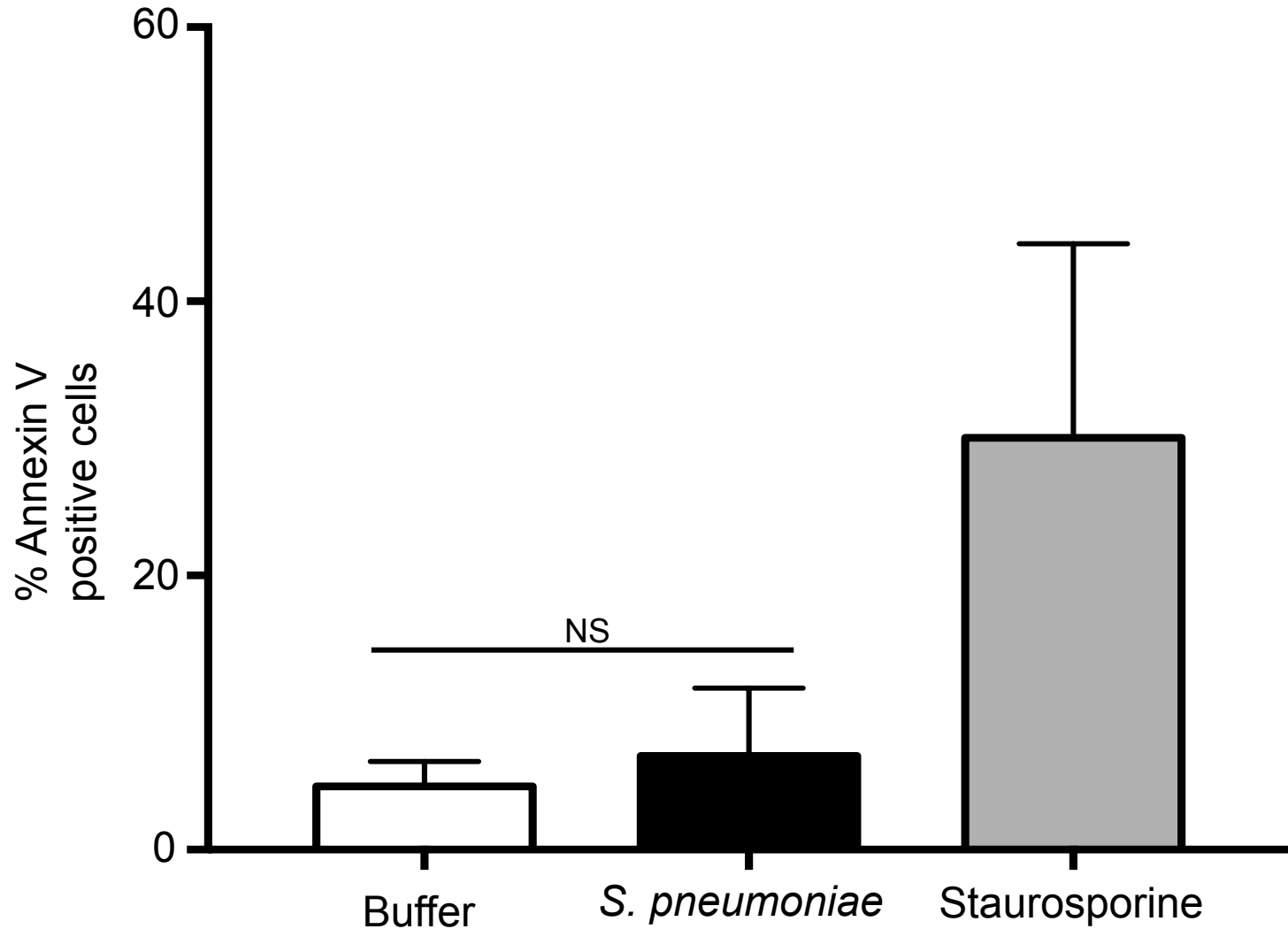

Supplement: FIG S2 [file sph003182582sf2.pdf]

# Supplemental Fig. 3

A.

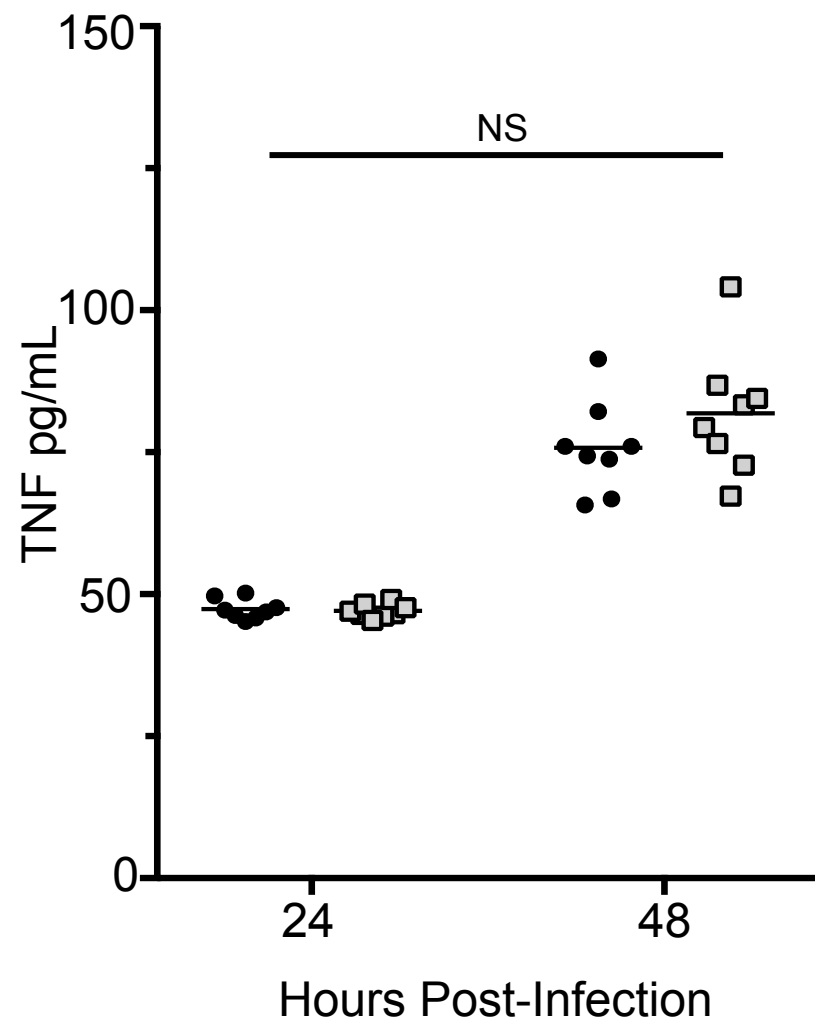

B.

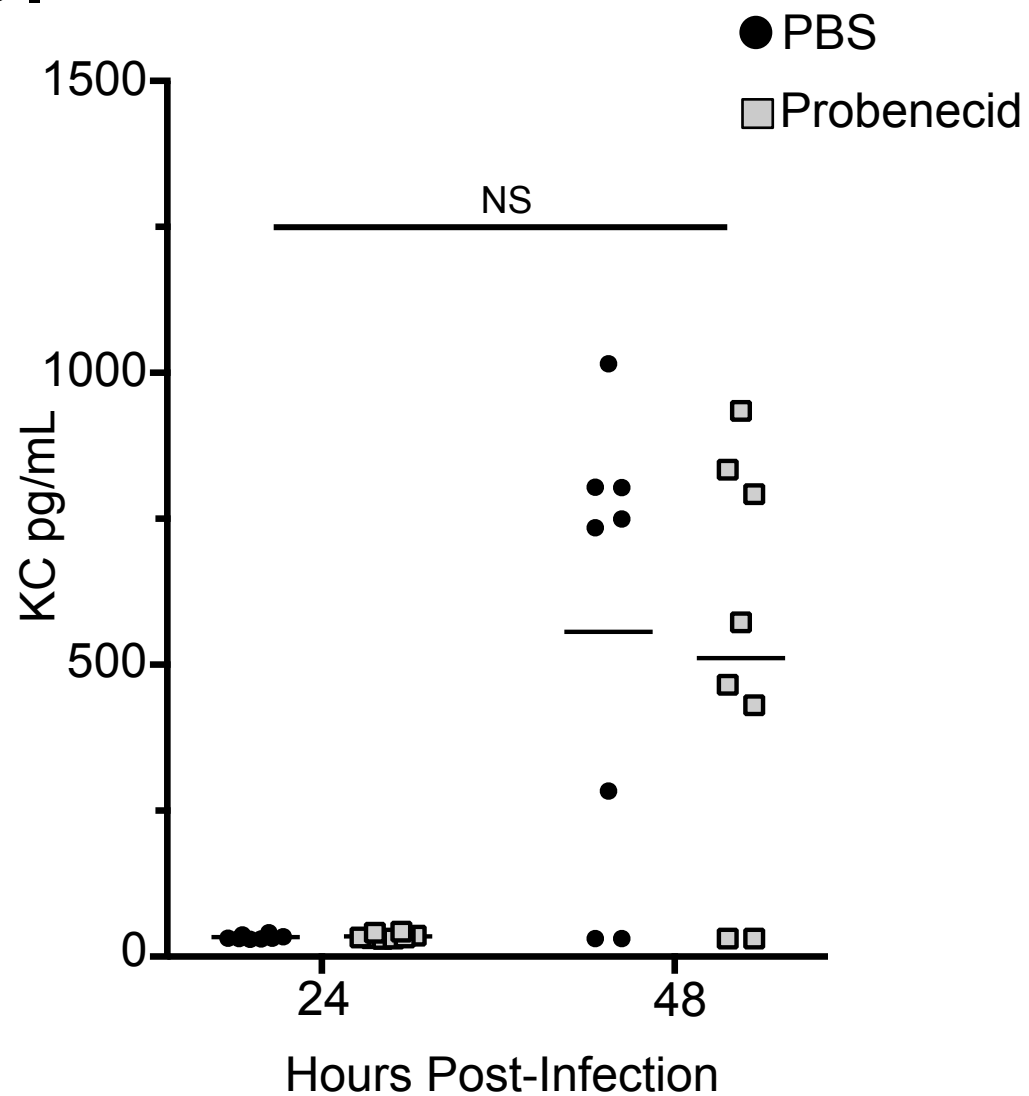

C.

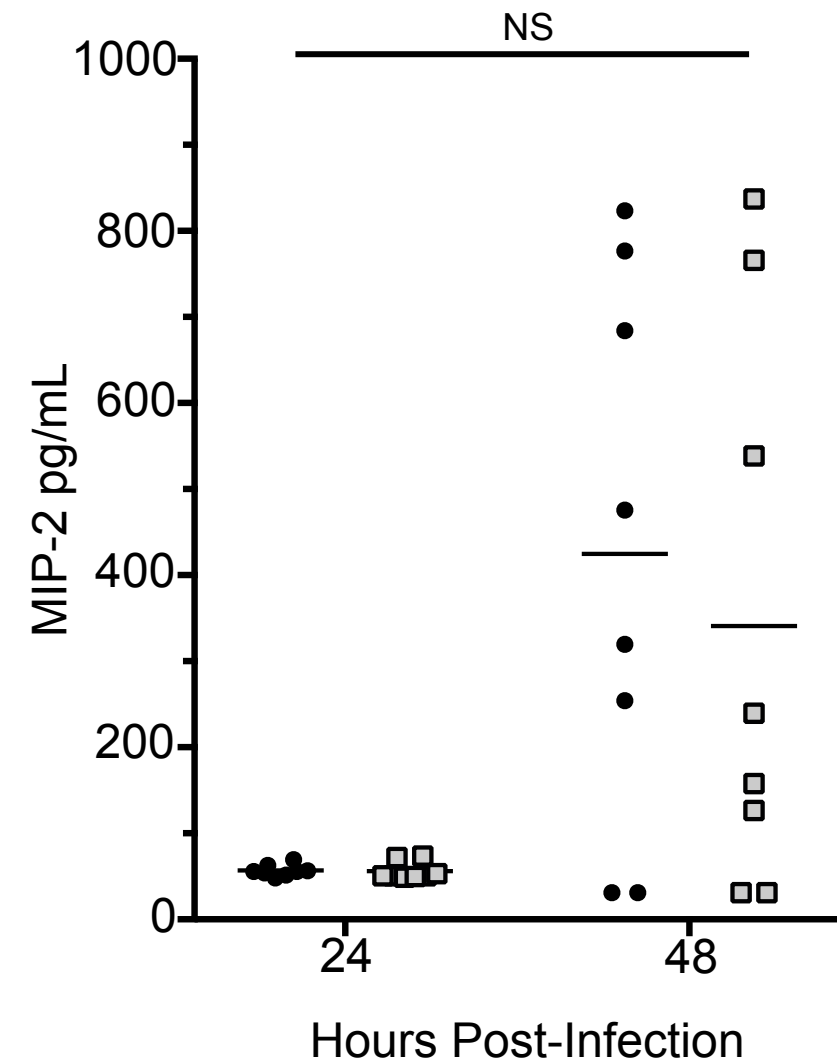

D.

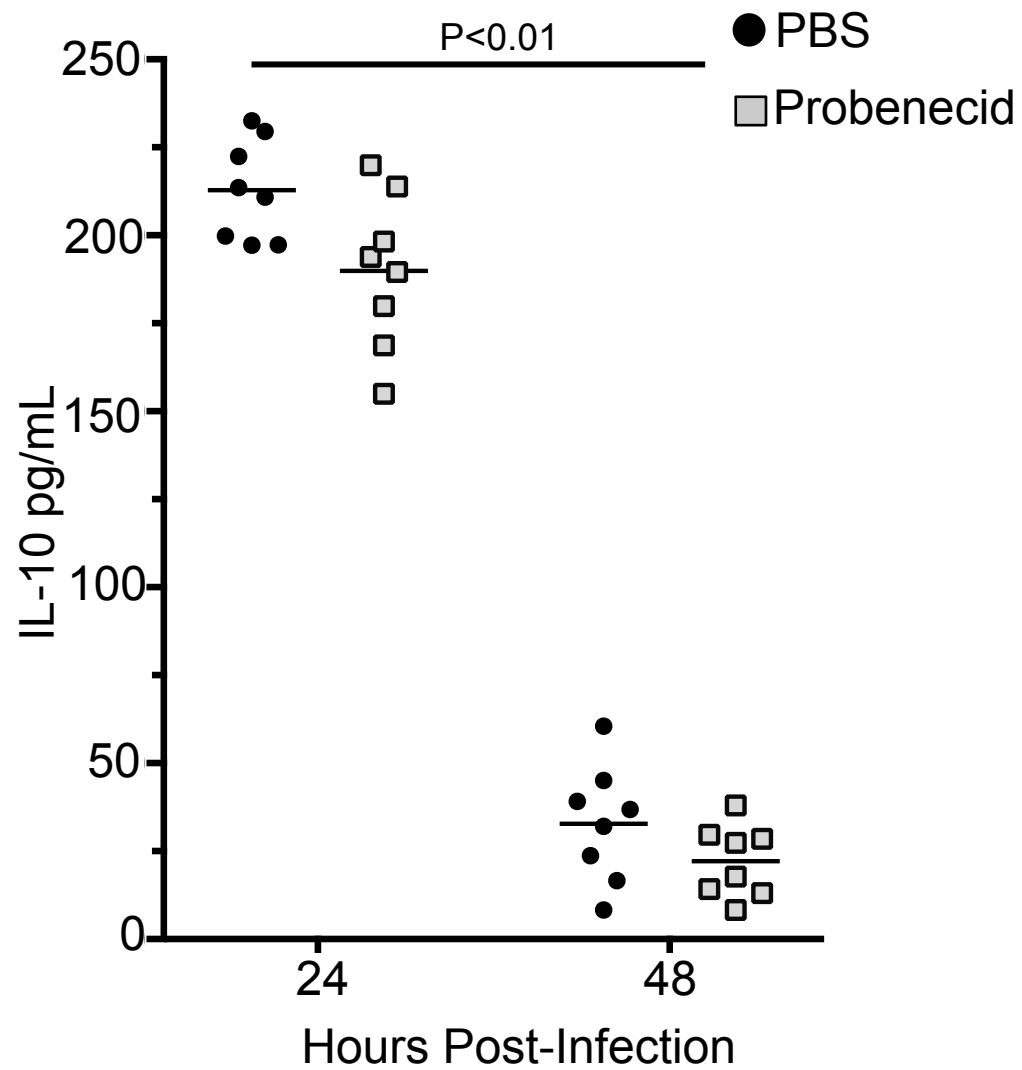

Supplement: FIG S3 [file sph003182582sf3.pdf]

# Supplemental Fig. 4

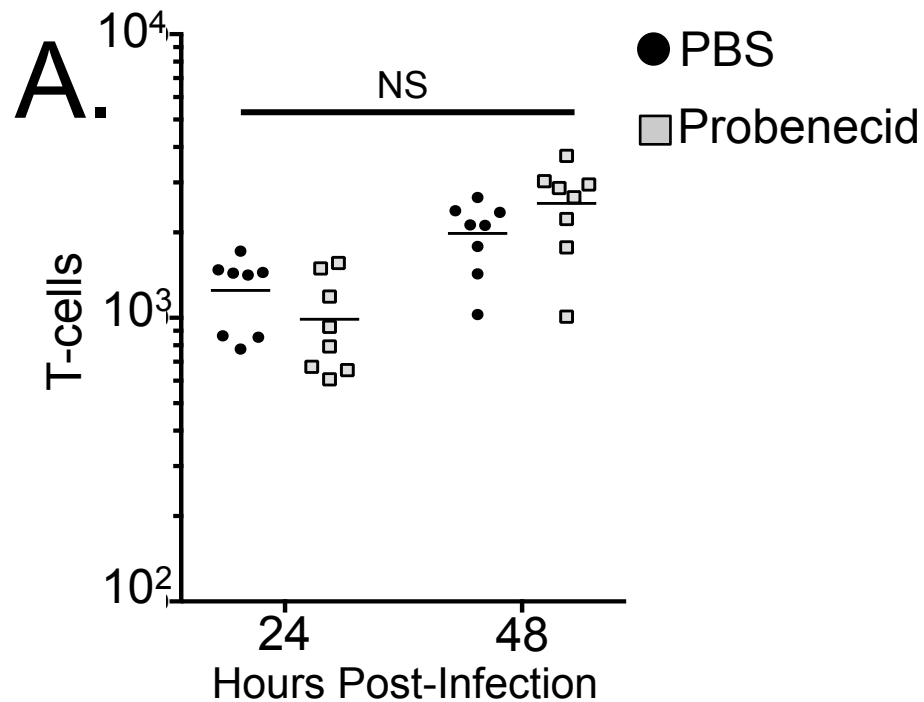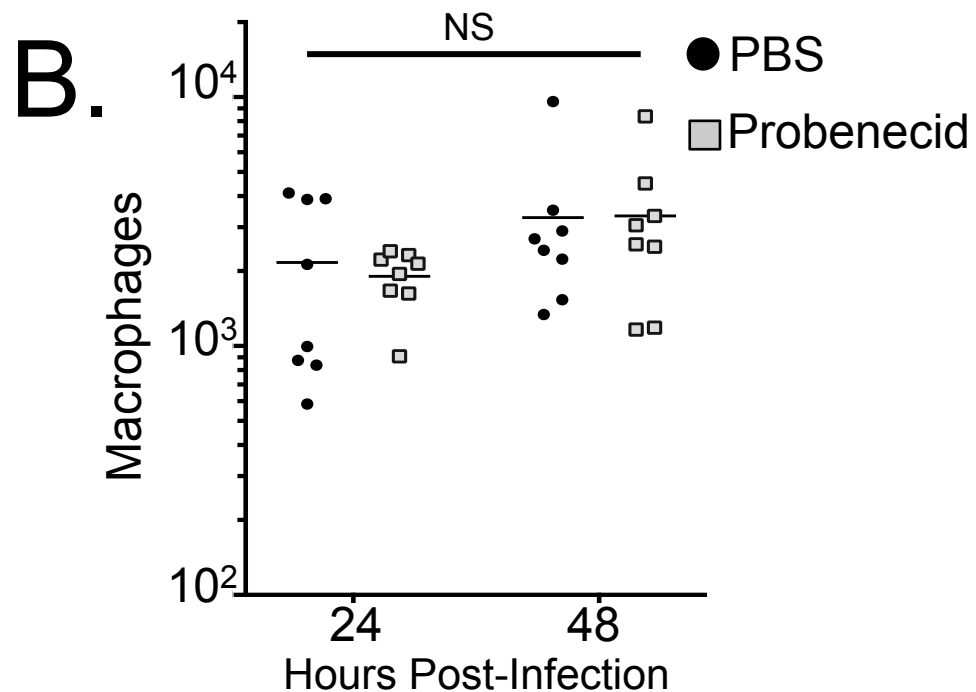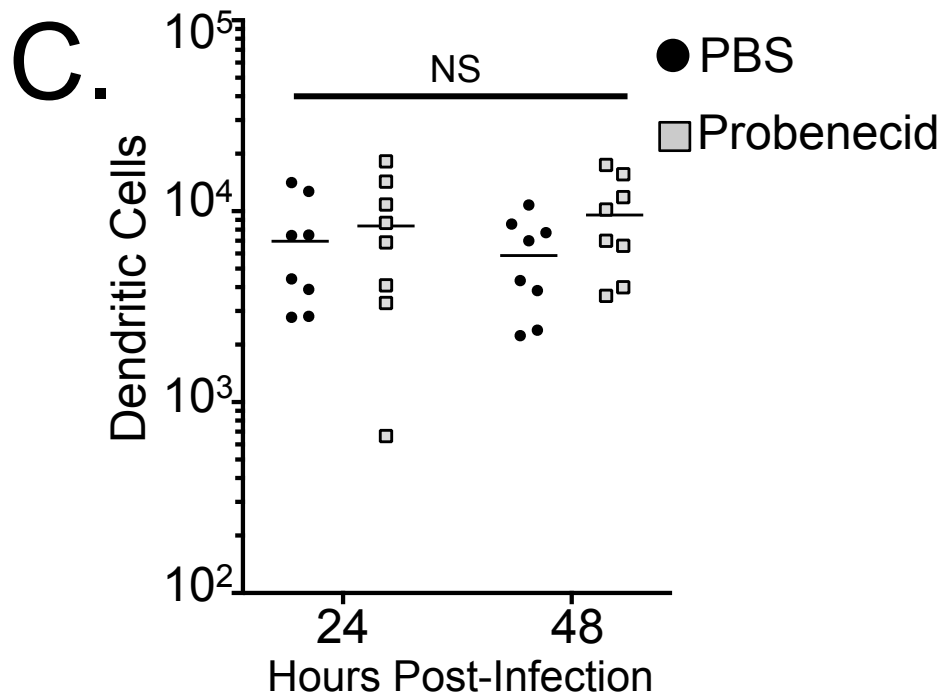

D.

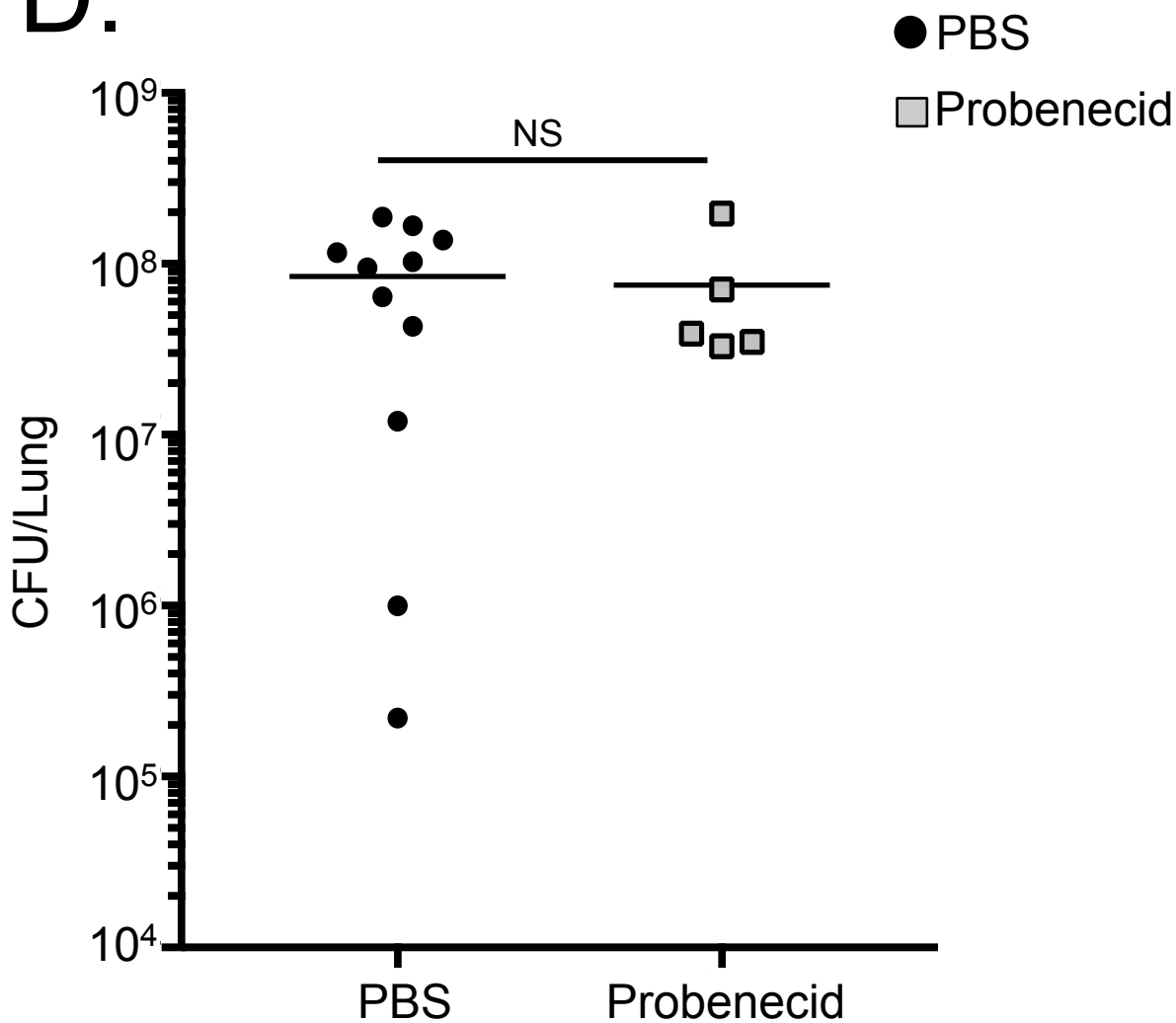

Supplement: FIG S4 [file sph003182582sf4.pdf]

# Supplemental Fig.5

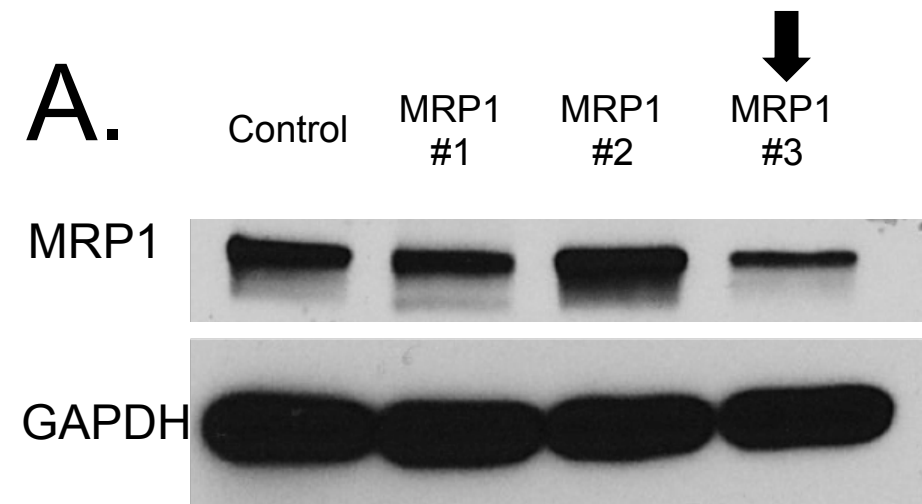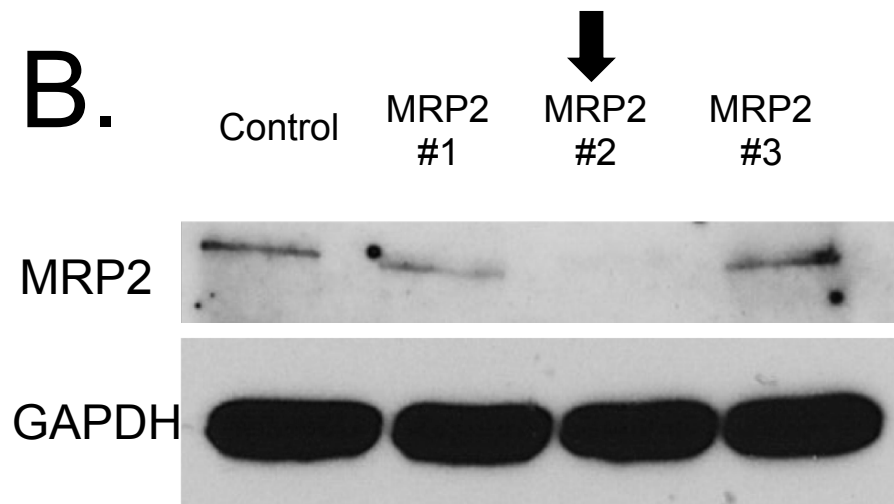

Supplement: FIG S5 [file sph003182582sf5.pdf]
